# Effect of simulation-based training workshop on obstetric emergency team collaboration and communication: a mixed study

**DOI:** 10.3389/fmed.2024.1282421

**Published:** 2024-03-20

**Authors:** Na Wu, Wei Li, Rong Huang, Hui Jiang

**Affiliations:** ^1^Nursing Department of Shanghai Key Laboratory of Maternal Fetal Medicine, Shanghai Institute of Maternal-Fetal Medicine and Gynecologic Oncology, Shanghai First Maternity and Infant Hospital, School of Medicine, Tongji University, Shanghai, China; ^2^Anesthesiology department, Shanghai Key Laboratory of Maternal Fetal Medicine, Shanghai Institute of Maternal-Fetal Medicine and Gynecologic Oncology, Shanghai First Maternity and Infant Hospital, School of Medicine, Tongji University, Shanghai, China

**Keywords:** midwife, simulation-based training, experience, team cooperation, obstetric emergency situations, clinical teamwork scale

## Abstract

**Aims and objectives:**

To explore the effects of simulation-based midwife training workshops and determine whether such a program can improve team collaboration and communication.

**Background:**

Simulation training improves communication, team cooperation, critical thinking, and situational awareness.

**Design:**

This mixed study was conducted September 15–18, 2021.

**Methods:**

Participants included 23 obstetricians and midwives who completed 2 days of simulation training, including communication, skills, teamwork, single technical operation, and scene running. The Clinical Teamwork Scale was used before and after the comparison, and the data were analyzed using a phenomenological analytic process.

**Results:**

The total team cooperation, transparent thinking, closed-loop communication, overall decision-making, clear responsibility, and leadership scores of the trainees were significantly higher after than before the training. The experience of attending a simulated training workshop can be divided into two themes: innovative ways of offering training and active learning. Three key themes emerged from each category: education combined with recreation; full participation in interactions; and teamwork and communication. (1) application of knowledge (2) dissemination, and (3) sublimation of knowledge.

**Conclusion:**

This study’s findings indicated a good experience and higher team cooperation score among midwives participating in simulation-based training in China, the value of our work is to show that the researched teaching methods, although published in other contexts, are also valuable in the Chinese context, suggesting that they will pass on the methods and concepts of the simulated training to others and change the current status of classroom teaching, which is its most meaningful practical training effect.

**Relevance to clinical practice:**

These results imply that simulation-based midwife training for obstetric emergencies is required to improve the comprehensive ability of midwives to address obstetric emergencies, thereby improving maternal clinical outcomes.

**No patient or public contribution:**

Neither patients nor the public were involved in this study, and the midwives and obstetricians voluntarily participated.

## 1 Introduction

The cognitive load (CL) theory was proposed by van Merriënboer and Sweller (1) and consists of the following (2): internal and extraneous loads of learning tasks and related loads of the learning process. In the CL theory, self-reported commitment and effort, stress levels, and dual-task performance can be estimated in different ways. However, intrinsic loads can be managed from simple to complex learning tasks and from low- to high-fidelity scenarios (1), which can be improved through simulation.

Simulation is considered an effective way to assess nurses (3), especially team performance. The goals of simulation-based training especially for teamwork are addressing roles, situational awareness, leadership, communication, and responsibilities (4). The modern model of surgical simulation training, also called simulation-based training (SBT), which allows surgeons to obtain clinical experience or operating room skills in the simulation environment, is increasingly emerging with technological development (5). Moreover, the Accreditation Council for Graduate Medical Education has approved and integrated SBT into surgical curricula (6).

In the light of medical errors being the third leading cause of death in America, after heart disease and cancer (7), we know that supporting others, solving problems, exchanging information, and leadership can reduce the effects of human error during the simulation training process (8). Simulation training is superior to lecture-based teaching of technical and nontechnical skills (9) in terms of competence, confidence, and communication. Adult education in the field of obstetrics and gynecology has gradually shifted from traditional approaches, including lectures and teaching in clinical settings, to simulation-enhanced education. Learning about pelvic examinations through simulation-enhanced education brings a significant benefit to medical and midwifery students, thereby improving their technical skills, comfort, and patient communication (10). The number of articles on simulations has rapidly increased and simulation-based multidisciplinary team training has become popular over the past few years. A study from Pakistan showed that team performance significantly improved after training (11), highlighting the need to teach communication and leadership skills through simulation workshops. Similarly, Grogan et al. (12) showed that training in crisis resource management generated a positive attitude among trainees regarding coping with exhaustion, team building, communication with each other, and situational awareness.

The use of simulation training has also spread rapidly in China over the past 3 years, especially in the obstetrics community. Currently, in Shanghai, almost every midwife and nursing training program is held in our simulation training center; thus, we must understand the effects of simulation training and receive participant feedback.

The launch of the three-child policy by the Chinese government has raised concerns about a shortage of midwives; in addition, there will be a higher proportion of older mothers with potential complications. The “three delays,” including in seeking appropriate care, obtaining timely care, and providing care, contribute to the pooled quality of services (13). Failures in communication, event reporting, and leadership are the root causes of patient safety incidents (14). These factors challenge the entire medical system and threaten maternal and child safety. Fostering trained midwives to adapt to different situations is important, and training may play an important role (15).

Few studies have explored midwives’ team collaboration and communication or their feelings regarding attending training, especially in China. Our simulation workshop emphasizes team building, communication skills (such as closed-loop communication), leadership skills, rescue processes, and situational awareness. Therefore, here we sought to understand the midwives’ thoughts and determine whether there was a difference between simulation- and lecture-based training. The nursing department of our hospital organized a national continuing medical education program called The Simulation-Based Training Workshop for Midwives. This study aimed to evaluate the effects of SBT programs on obstetric emergencies.

## 2 Materials and methods

### 2.1 Study design and settings

This study utilized a quasi-experimental and comparison group research design. A workshop that enrolled 24 participants was held at the simulation center of our hospital, which has two campuses and manages nearly 30,000 births annually.

### 2.2 Study participants

Midwives and obstetricians were eligible to attend the simulated training until the quota of applications was full upon meeting the following criteria: (1) worked in the labor room; and (2) spoke Mandarin. Table 1 shows the participants’ baseline data.

**Table 1:** Professional and educational characteristics of study participants.

Age, years	28.69 (21–42)
Sex	
Female	21
Male	2
Profession	
Midwife	20
Obstetrics resident	3
Hospital	
Tertiary general hospital	7 (30.4%)
Secondary general hospital	7 (30.4%)
Tertiary specialized hospital	7 (30.4%)
Private hospital	2 (8.0%)

Values are shown as median (interquartile range), *n*, or *n* (%) as appropriate.

One obstetrician was unable to participate due to work conflicts. Thus, a total of 23 participants, including 20 midwives and three obstetricians, participated in the simulated training. Sixteen midwives were interviewed because no new subject words appeared and the interview data were saturated. None had previously participated in SBT. This study was approved by the Ethics Committee of the Hospital, and the number was KS2026. All participants provided written informed consent before participating.

### 2.3 Simulation workshop

The SBT workshop was conducted September 15–18, 2021. The study was conducted at the simulation center of the hospital, which featured high-tech simulation equipment such as a SimMother, New B of Laerdal, and a Noelle™ birthing simulator (Gaumard, Miami, FL, United States). High-fidelity simulators produce respiratory sounds, heart sounds, and palpable pulses and are equipped with a monitor that displays the electrocardiograph, fetal heart rate, blood oxygen saturation, blood pressure, arterial waveform, pulmonary artery waveform, and anesthetic gas. Moreover, these simulators can be combined with computer simulations to conduct multidisciplinary team crisis management training.

**Figure 1:**
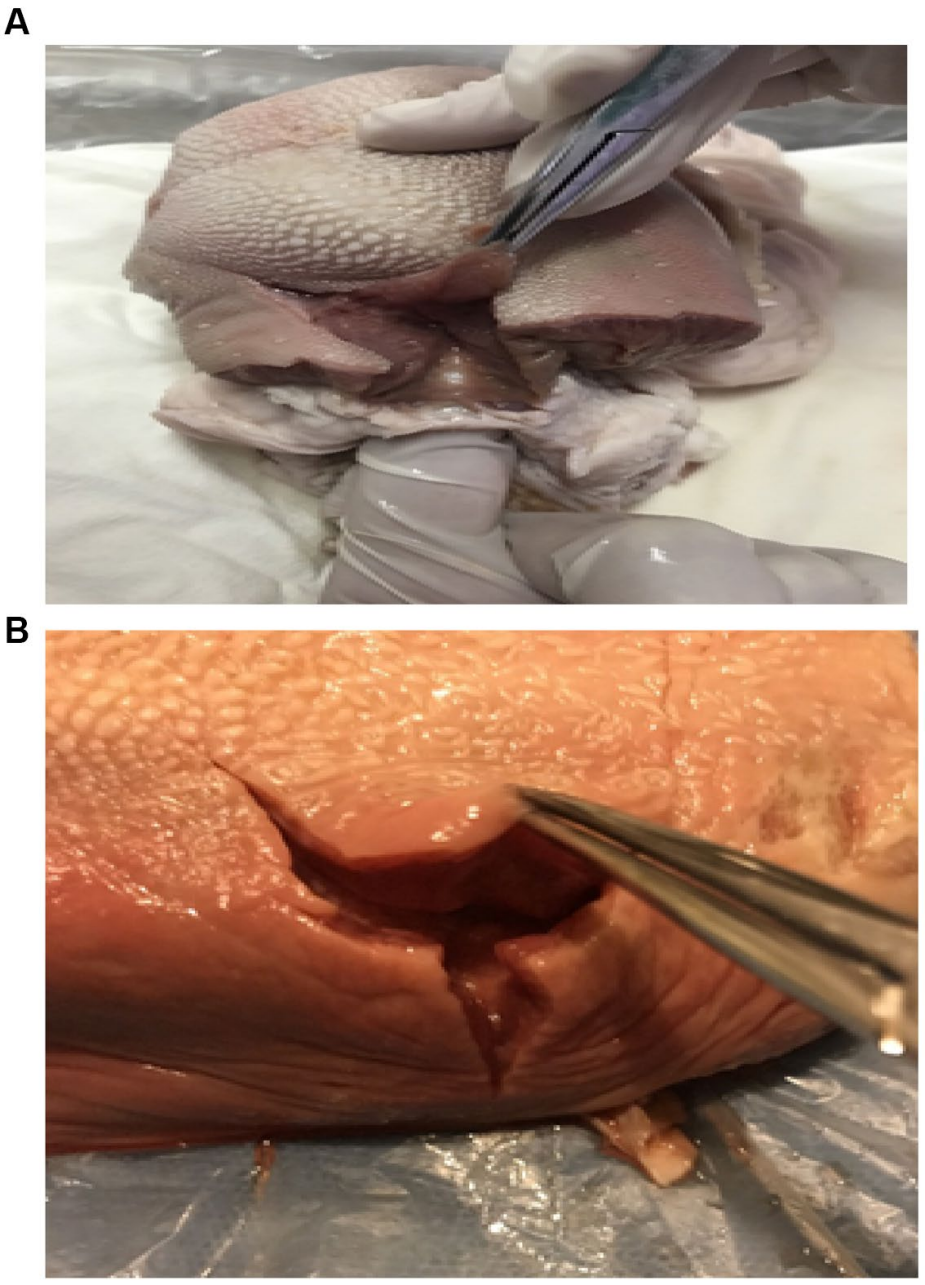
**(A, B)** Perineal wound using a cow’s tongue.

The workshop included training interventions using computerized simulators and high-tech mannequins. The participants were separated into groups of 4–6 according to different scenarios. For example, the postpartum hemorrhage scenario featured six roles; thus, the participants were separated into four teams, and we ran the postpartum hemorrhage scenario four times. The participants in each team were separated by hospital level and working age until the groups were balanced. Each was an active participant and took turns in different scenarios, including the following: gentle birth, episiotomy model of the perineum (Figures 1A,B), shoulder dystocia, neonatal asphyxia resuscitation, emergency cesarean section, postpartum hemorrhage, and preeclampsia with seizures. The trainers introduced the environment and equipment before starting each scenario.

Before the simulation workshop began, all programmed scenarios were tested at least three times. The bedside monitor displayed the vital signs during the simulated scenarios. The team members must focus on and act on physiological data from the monitor, which was operated by an experienced faculty member in the tiring-room. Each scenario lasted for 15 min and was followed by a 20 min debriefing session. Topics included teamwork, communication skills, and leadership, all of which are important elements of an effective team in obstetric emergencies. During the debriefings, the video was suspended when the facilitators asked the participants to discuss and reflect on their performances.

**Figure 2:**
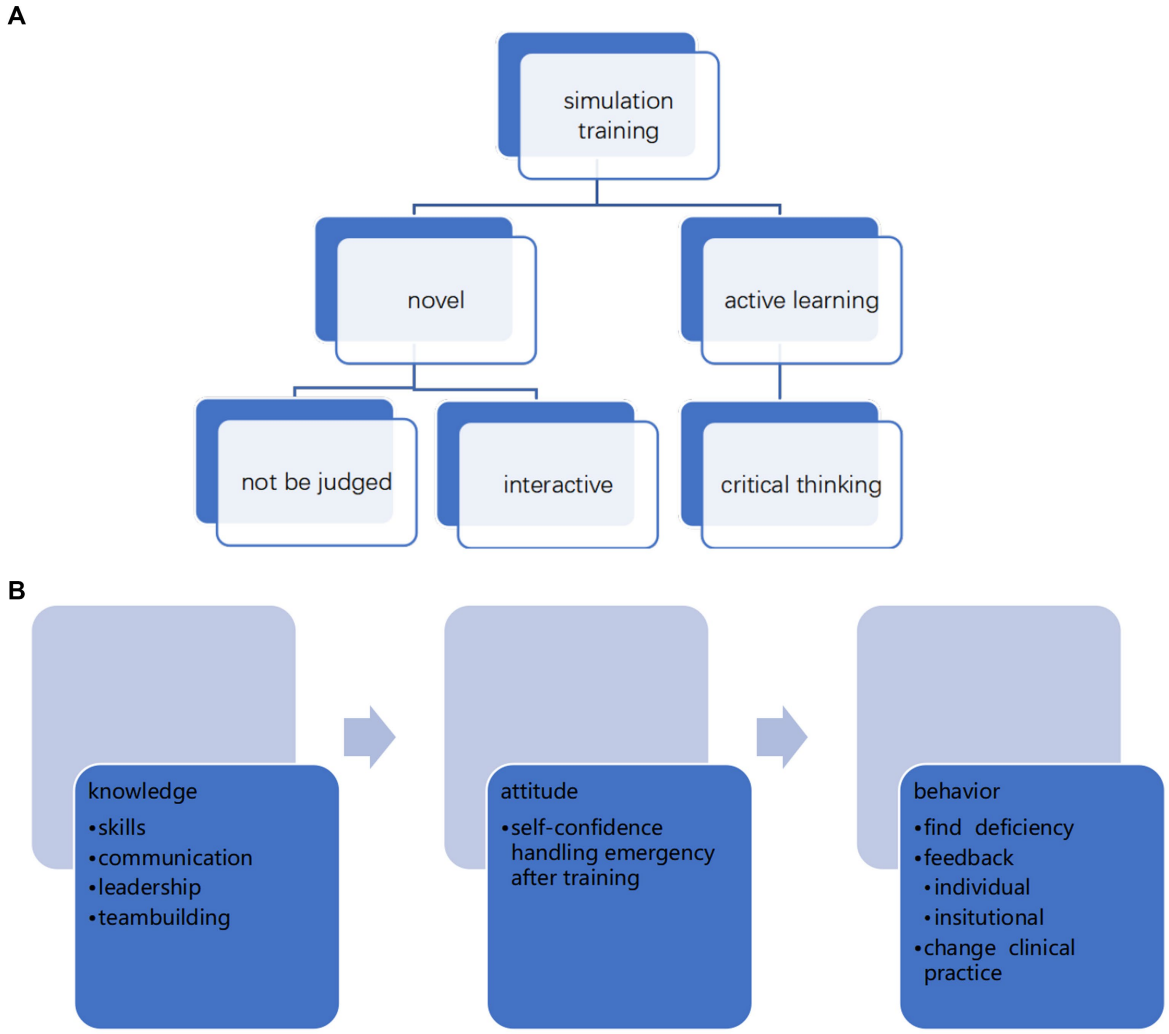
**(A, B)** Midwives’ experiences.

We hypothesized that SBT would improve teamwork performance, especially communication skills, and enhance the training experience (see Figure 2).

### 2.4 Data collection procedure

The Clinical Teamwork Scale (CTS) was used to assess team performance in a simulation-based obstetric emergency context (16). The CTS has reported substantial agreement (kappa, 0.78) and high inter-rater reliability (interclass correlation coefficient, 0.98) (17) and contains teamwork behaviors that are applicable to any resuscitation environment. The CTS includes an overall/global teamwork rating as well as ratings for elements within five teamwork domains: communication, role responsibility, situational awareness, decision-making, and patient friendliness (all scored on a 10-point Likert scale). Item 9 is target fixation, which refers to the fact that in a simulated scene, the trainees are subjected to tunnel vision, which causes obstacles to management of the entire scene (18). This scale evaluates team performance in obstetric emergency simulations. The operational scenarios in our training workshop covered obstetric emergencies and dangerous situations; therefore, this scale was used to evaluate team cooperation.

### 2.5 Data management and analysis

A blinded review of the participants’ performance in these scenarios was conducted by two trained nurses, one with expertise in emergency obstetrics and the other with experience in simulated education. An audit of 25% of the 32 scenarios demonstrated 99% consensus between the two reviewers.

### 2.6 Statistical analyses

The data were analyzed using Statistical Package for Social Sciences (version 26.0; IBM Corp., Armonk, NY, United States). CTS scores are described as median (range). To compare the differences in team performance between the pre- and post-training groups, the total median and median score of each CTS item were calculated, and a signed-rank test was conducted to investigate the difference in CTS scores. Statistical significance was accepted at a two-sided value of *p* < 0.05.

After the workshop, two researchers (NW and HJ) conducted in-depth interviews with 16 midwives that began with open-ended questions. An in-depth semi-structured interview outline was used (Table 2). The participants’ behaviors and emotions were also noted. Each interview, which was conducted in a separate room, lasted 15–20 min. Body language was also noted to facilitate a later data analysis.

**Table 2:** Semi-structured interview guide.

Questions
1. Have you heard of simulation training before?
2. Have you participated in obstetric emergency training before?
3. Why are you attending the simulation training?
4. Can you talk about your feelings about the simulation training?
5. Is there a gap with your expectations? If so, how?
6. What is the biggest impact of participating in simulation training on you?
7. How will your work change after this simulation?
8. Is there anything else you would like to share with us?

Colaizzi’s (19) seven-step framework was used for the data analysis. To divide the experiences into categories, a passage reading was first performed within 24 h after the interview, and each participant’s experience was read and assigned a keyword. The keywords were collected in tabular form. At the same time, we assigned every experience a number, so we could note the number and keyword of the experience in a second table and retrace how each experience was coded later. To combine the collected codes into supercategories, it was necessary to consider which keywords were similar or could be condensed and attributed to a similar behavior. In the final step, it was possible to assign all experiences to different categories. The results were cross-reviewed by two investigators and any disagreement was resolved by discussion with a third investigator. The transcribed data and extracted themes and sub themes were sent back to the participants and to ensure that the findings reflected their actual perspectives rather than the investigators’ understanding of the phenomenon, a follow-up conversation was conducted.

After the workshop, the SBT was evaluated using a self-designed questionnaire, of which the content validity was mainly calculated using five expert scores, and the content validity index was 1. The questionnaire consisted of 17 workshop items that were answered using a five-point Likert-type scale (Table 3).

**Table 3:** Questionnaire including 17 workshop-related items.

*N* = 23	Item	Mean ± SD
Training objectives	1. The teaching goals are clear, reasonable, and practical	4.67 ± 0.48
	2. The objectives meet the needs of clinical work	4.67 ± 0.48
Simulation environment	3. The preparation matches the scenario description	4.67 ± 0.48
	4. The layout of the scene is reasonable	4.70 ± 0.46
Training content	5. There is plenty of information about the simulation scene	4.75 ± 0.44
	6. The scenarios are instructive and scientific	4.70 ± 0.46
	7. The theory is linked with practice	4.75 ± 0.44
	8. The simulation increased the knowledge from books	4.70 ± 0.46
Training methodology	9. The method is novel and interactive	4.79 ± 0.41
	10. The scenarios are scheduled for a reasonable time	4.79 ± 0.41
	11. Simulation is more useful to me than traditional lectures	4.79 ± 0.41
Training tutor	12. The tutors make me feel relaxed	4.88 ± 0.34
	13. The tutors are instructive	4.92 ± 0.28
Training application	14. The training content can be applied to my future work	5.00 ± 0.00
	15. The simulation training will be helpful to my work	4.96 ± 0.20
	16. Simulation training will be helpful to my teamwork abilities	4.96 ± 0.20
	17. Simulation training will be helpful to my communication abilities	4.96 ± 0.20

## 3 Results

### 3.1 Team training was associated with higher median CTS

The total median score of all items was significantly higher in the post-training group (median, 7.0 range, 4.0–9.0) than in the pre-training group (median, 5.0; range, 4.0–6.0; *p* = 0.002). A comparison of the median CTS scores for communication, transparent thinking, overall situational awareness, and leadership showed a statistically significant difference between the pre- and post-training groups (Tables 4, 5).

**Table 4:** Comparison of team performance pre- and post-simulation training assessed by the Clinical Teamwork Scale.

Item	Post-training	Pre-training	Statistic	*p*-value
1. Overall score	7.0 (4–9)	5.0 (4–6)	3.115	0.002
2. Overall communication	7.0 (5–9)	5.0 (3–6)	3.370	0.001
3. SBAR	7.0 (5–9)	4.0 (3–6)	3.783	0.000
4. Transparent thinking	7.0 (5–9)	6.0 (4–8)	2.410	0.016
5. Directed communication	7.0 (5–9)	5.0 (2–6)	3.313	0.001
6. Closed loop	7.0 (5–9)	5.0 (2–6)	3.552	0.000
7. Overall situational awareness	7.0 (5–8)	6.0 (4–8)	2.401	0.016
8. Resource allocation	6.0 (5–7)	6.0 (3–8)	0.722	0.470
10. Overall decision making	6.0 (5–8)	6.0 (4–8)	0.741	0.458
11. Prioritizing	6.0 (5–8)	6.0 (3–8)	1.384	0.166
12. Overall role responsibility	6.0 (5–8)	6.0 (4–8)	1.904	0.071
13. Role clarity	6.0 (5–9)	6.0 (4–8)	1.709	0.088
14. Perform as a leader/helper	7.0 (5–9)	5.0 (4–7)	3.438	0.001
15. Patient friendly	7.0 (4–9)	6.0 (4–8)	1.897	0.058

SBAR, situation, background, assessment, and recommendation.

**Table 5:** Comparison of presence of target fixation (item 9 of Clinical Teamwork Scale) pre- versus post-simulation training.

Item 9	Post-training (*n*)	Pre-training (*n*)	Total
No target fixation	10	7	17
Target fixation	2	4	6
Total	12	11	23

### 3.2 General characteristics of interviewed study participants

In our study, the 16 midwives were 22–46 years of age, they came from different provinces of China, and their education levels ranged from junior college to university. Table 6 summarizes the characteristics of each sample.

**Table 6:** Characteristics of interviewed midwives.

Code	Age	Education level	Hospital	Work experience, years
1	36	Bachelor’s degree	Tertiary general hospital	11
2	25	Bachelor’s degree	Secondary general hospital	3
3	28	Bachelor’s degree	Tertiary specialized hospital	2
4	29	Bachelor’s degree	Tertiary specialized hospital	2
5	25	Associate’s degree	Tertiary general hospital	4
6	28	Associate’s degree	Tertiary general hospital	4
7	23	Bachelor’s degree	Tertiary specialized hospital	2
8	22	Bachelor’s degree	Tertiary general hospital	6
9	22	Associate’s degree	Tertiary general hospital	2
10	32	Bachelor’s degree	Secondary general hospital	6
11	31	Bachelor’s degree	Secondary general hospital	10
12	21	Bachelor’s degree	Tertiary general hospital	1
13	33	Associate’s degree	Tertiary general hospital	3
14	33	Associate’s degree	Tertiary general hospital	2
15	26	Bachelor’s degree	Tertiary general hospital	1
16	27	Associate’s degree	Tertiary general hospital	3

### 3.3 Effectiveness evaluation by midwife staff after training with face-to-face interviews

Satisfactory reactions emerged from the in-depth interviews. The experiences of midwives participating in the SBT can be summarized into two themes: novel training methods and active learning processes.

#### 3.3.1 Midwives’ feelings about novel training methods

In traditional Chinese teaching and training, students are often judged based on their grades, and trainees are frequently criticized. Under huge psychological pressure, most are unwilling to participate in training; rather, they passively accept the offered knowledge. SBT aims to identify and solve problems and improve capacity rather than criticize or blame. Therefore, midwives who participated in this SBT experienced new feelings.

#### 3.3.2 Simulation training combines teaching and enjoyment

During the scenarios, the trainees interact with each other and the trainer provided a visual display of a standardized patient, especially when “family members created trouble,” which made the training impressive.

*Case 1*

*The training mode is very new. I only learned about this learning mode from textbooks before. I didn’t think I would have the opportunity to experience such training after work*.

*Case 4*

*I didn’t care much about patient’s family psychology in the past, but the standardized patient gave me a significant impression of the patient’s perception of care. When we tried to save the women, her husband continued to ask what happened to his wife. When you participate in a lecture, the teacher would just tell you, “Comfort the family,” but then you may forget; however, after this simulation training, I would not*.

##### 3.3.2.1 Simulation training combines teaching with interaction

Traditional teaching involves teachers lecturing and students listening. For adult teaching, it may be difficult to stimulate a student’s motivation to participate in the learning process, and the effect of training remains to be discussed.

*Case 2*

*This model is vivid, which allows us to participate. It is more memorable than the traditional teaching model*.

*Case 3*

*I felt that all of the tutors were very professional and enthusiastic with close interactions. I learned a lot*.

*Case 6*

*I would listen to the tutors because they show how to do something and why. I didn’t get bored and doze off*.

*Case 9*

*I learned “press the pubic symphysis” from a lecture and textbook about shoulder dystocia, but I didn’t have the chance to operate the procedure. In the simulation training, I performed it, and the tutor provided advice. We interacted and I was given feedback*.

Simulation training focuses more on student participation, arouses their enthusiasm, and encourages them to perform more work. Teachers play a guiding role and complete the teaching objectives by inspiring learning.

#### 3.3.3 Simulation training emphasizes teamwork and communication

Workers in the labor room are not individuals; rather, they comprise a multidisciplinary team. Students further understood the importance of teamwork and communication through obstetric emergency simulation training.

*Case 15*

*In the simulation scenario, we are required not only to form a group but also to communicate and cooperate with each other, which we usually would not be taught using books and lectures*.

*Case 7*

*Members conduct necessary action coordination, and a leader developed the team’s ability to solve problems by relying on teamwork and communication*.

*Case 11*

*We just kept silent during the first scenario and did our own work, which was strange …After this workshop, I know we are all small building blocks in our work. Only by cohesion can we build a wonderful world*.

#### 3.3.4 Midwives’ feelings about active learning process

##### 3.3.4.1 Simulation training learning and application of knowledge

After the simulation training, students can take initiative to compare the results with their previous work styles and improve. These areas range from skills to theory to teamwork.

*Case 2*

*I will bring your new ideas to our hospital so that we can help the women*.

*Case 4*

*When I was a doula, I wanted the women to give birth as soon as possible. I didn’t pay attention to their psychology or comfort, which I will pay more attention to now*.

*Case 8*

*I used to aim to do my own work well. Now (after training), I know to cooperate and communicate with others as a team*.

##### 3.3.4.2 Simulation training learning and spread of knowledge

After the training, students not only changed the way they worked but also shared valuable lessons with their colleagues. This reflects an active learning process.

*Case 3*

*I feel that your equipment and concepts are very advanced. I would like to give our leader some feedback after returning to work. Perhaps the hardware was not the same as yours, but we can change the concept step by step (laughter)*.

*Case 5*

*A doctor in our hospital performed a cesarean section when the fetal heart slowed rather than attempting intrauterine fetal resuscitation. I would like to provide feedback when I return*.

##### 3.3.4.3 Simulation training turns knowledge into value

The trainees recognized the interesting methods and effects of simulation training and expected to obtain further learning and more training opportunities to constantly improve their abilities. Some were even planning to pursue teaching as part of their career path.

*Case 10*

*I hope to learn more obstetric emergency training, such as cardiac arrest and amniotic fluid embolism*.

*Case 15*

*I hope to have the opportunity to participate in training frequently so that when I am old (working years and seniority promotion), I can also train new midwives (laughter)*.

*Case 16*

*Your training method impressed me so much that I will try it with our younger (junior) midwives after I return*.

### 3.4 Evaluation of satisfaction degree of midwife staff after training using self-assessed questionnaire

The mean scores for training objectives, simulation environment, training content, training methodology, training tutors, and training applications are listed in Table 3. All interviewees enjoyed the simulation-based midwifery training workshop and hoped to participate in it in the future. They believed that team building and cooperation, closed-loop communication, and situational awareness could be learned through simulation training.

All participants were interested in the workshop and gained new knowledge. As Pacheco Granda and Salik (3) demonstrated, our course design ensured that the materials and equipment in these scenarios were realistic and appropriate for clinical practice with sufficient physiological complexity.

## 4 Discussion

This study aimed to describe midwives’ attitudes, experiences, and team performance in SBT workshops in China. We found that simulation training allowed students to fully participate in operations, which can improve their skill levels, knowledge, and team cooperation scores compared with purely theoretical teaching. These findings contribute to the knowledge that trainees, especially midwives, have low seniority. Many changes in their perceptions have improved their ability to think critically and transparently. This is in line with previous evidence that, during simulated scenarios, teams could practice, reflect, and provide solutions to potential obstacles to medical care (20). Simulation training has been shown in improving knowledge acquisition (3). Moreover, participants could perform these operations on the simulator, which provided tools that allowed them to bridge theory and practice (21). This study also indicated a good experience with SBT, as participants could practice repeatedly in a familiar and safe environment without patient harm (4).

Our study showed a higher team cooperation score in the post-training group compared to the median scores in the pre-training group. Because the simulation-based team training emphasized effective communication, team cooperation, leadership, and situational awareness. This was also in line with Chang’s study (22), which reported a significant difference between simulation and lecture training in total Situation Awareness Global Assessment Technique scores, and situational awareness increased mainly because of the improvement in perception abilities.

Simulation programs reportedly improve safety and communication skills (23). Thus, simulation training made the participants feel safe and they would not be judged to the same criticism as they would in traditional training. According to the CL theory, in low- and high-fidelity environments, intrinsic loads can be managed, relaxing participants and allowing them not to worry about the task itself. When adults see the value and relevance of what they learn, they spend more time learning the subject. Otherwise, they need opportunities for feedback and reflection to ensure self-improvement (24), which could be reduced in simulation training. As previous studies (25, 26) demonstrated, simulations change midwives’ behaviors.

During SBT, debriefing, which is defined as feedback and a reflective response in a scenario in which participants discuss and reflect on their performance under tutor guidance, is considered the “heart and soul” link (27). First, participants were asked to briefly summarize what they had learned after running the scenario and then focus primarily on what had worked well and what could be improved rather than criticizing those things that had not gone well. Video playback played an important role in the simulations. During debriefing, the participants could see how they performed rather than how they thought they performed (28) or how things should be done according to routine rules.

Simulation training aims to identify and solve problems rather than criticize or place blame. Scores are the only criteria used in China’s traditional teaching and training programs, which trainees are more likely to be criticized and are under great psychological pressure. Most are unwilling to participate in training; therefore, a safe environment should be provided (29). Trainers should create a “safe” training environment free from psychological burdens that allows trainees to actively and voluntarily participate in simulated training.

Through simulation training, midwives, especially those with low seniority, can master basic theories, knowledge, and skills in clinical work along with good communication and emergency handling abilities. These midwives can provide standardized services for patients to prevent and manage obstetric emergencies (30) and improve the clinical outcomes of pregnant women.

Most of the findings are well known in the world literature, however, the value of our work is to show that the researched teaching methods, although published in other contexts, are also valuable in the Chinese context. This enhances the validity of the methods. Our workshop was more like “training the trainers,” when they returned to their respective units, students not only changed the way they worked but also shared some valuable with colleagues. They passed on the methods and concepts of simulated training and changed the current status of classroom teaching, which was the most meaningful practical training effect.

### 4.1 Study limitations

This study was conducted at the largest obstetrics and gynecology simulation center in Shanghai, China. Participants from the simulation training workshop comprised different levels of midwives from different hospitals throughout the country. As a qualitative study, there were very few thoughts from doctors, and we must increase the number of obstetricians and assess their views on simulated training and the training effect in future studies. They established temporary teams to protect women from postpartum hemorrhage, shoulder dystocia, etc. The team members were unfamiliar with each other, although they completed a 2 days partnership. This may have affected team performance, particularly on the first day.

### 4.2 Relevance to clinical practice

These results suggest that simulated training can improve the training effect more than traditional lectures. The experience of attending simulated training includes innovative ways of offering training and active learning. The simulation training made the participants feel safe and not judged as by traditional teachers. This reduces the cognitive load. Thus, the environment was relaxed, the experience was new and innovative, and the scenario setting and teaching methods demonstrated by the tutors were full of heuristic. According to the knowledge-attitude-behavior pattern, good experience (knowledge gained in an interesting way) changed traditional learning attitudes and prompted the participants to reflect and make changes to their clinical practice. Our research explored only the experience of simulation training and team building, satisfaction with training, and improvement in trainee knowledge due to learning. In the future, we will study high-level simulation training, such as level three, referring to the application of learned behaviors and skills in clinical obstetrics practice. The fourth level refers to the effects of training on measurable clinical outcomes. Emergency obstetrics training must be performed using simulations that are safe for both healthcare providers and patients. The participants could perform repetitive operations, especially those related to rare emergencies, on a simulator without harming actual patients.

## 5 Conclusion

This study indicated a good experience and a higher team cooperation score of midwives participating in SBT in China. The experience completing the simulated training included innovative ways of offering training and active learning, passing the methods and concepts of simulated training on to colleagues, and changing the current status of classroom teaching, which is the most meaningful practical training effect. These findings imply that simulated training for obstetrics emergencies can be used to improve performance of both technical and nontechnical skills of midwives.

## Data availability statement

The raw data supporting the conclusions of this article will be made available by the authors, without undue reservation.

## Ethics statement

The studies involving human participants were reviewed and approved by the Ethics Committee of Shanghai First Maternity and Infant Hospital (Approval number: KS2026, Chairperson, Ye Luo). The patients/participants provided their written informed consent to participate in this study.

## Author contributions

NW: Writing – original draft, Formal analysis, Data curation, Conceptualization. WL: Writing – original draft, Supervision, Software, Conceptualization. RH: Writing – review & editing, Software, Methodology. HJ: Writing – review & editing, Supervision.
